# A rare case of bipartite combined tumour of the oesophagus

**DOI:** 10.1186/s12957-019-1623-7

**Published:** 2019-05-06

**Authors:** Nicholette Goh, Danson Xue Wei Yeo, Sanghvi Kaushal Amitbhai, Myint Oo Aung, Yong Howe Ho, Aaryan Nath Koura, Jaideepraj Rao

**Affiliations:** 1 Department of General Surgery, Tan Tock Seng Hospital, 11 Jalan Tan Tock Seng, Singapore, Singapore; 2 Department of Pathology, Tan Tock Seng Hospital, 11 Jalan Tan Tock Seng, Singapore, Singapore

**Keywords:** Oesophageal cancer, Small cell carcinoma, Combined tumours

## Abstract

**Background:**

Bipartite combined oesophageal tumours are an exceedingly rare entity and much less is known about the natural history of these tumours following curative surgery. The authors present a case of a bipartite combined oesophageal tumour comprising of sarcomatoid carcinoma and small cell carcinoma with early postoperative recurrence.

**Case presentation:**

A 63-year-old Chinese male with a smoking history presents with hemoptysis on a background of dysphagia and odynophagia for 1 month. An endoscopic evaluation found an exophytic oesophageal tumour with contact bleeding for which biopsy of this lesion returned as a malignant high-grade tumour where immunohistochemistry staining was unable to establish the lineage of the tumour. Differential diagnoses include sarcomatoid carcinoma and malignant undifferentiated sarcoma. With the provisional diagnosis of a high-grade oesopheageal sarcoma, the patient underwent minimally invasive McKeown’s oesophagectomy. Final histological assessment was pT1bN0 with two histological types of malignancy within a single tumour—70% poorly differentiated spindle cell squamous carcinoma and small cell carcinoma. He was planned for adjuvant chemotherapy in view of the small cell carcinoma component after the resolution of the postoperative infective collections. A computed tomographic scan performed 4 months postoperatively demonstrated metastasis to the lung, pleura, thoracic nodes and liver. Biopsy of the largest lung nodule confirmed small cell neuroendocrine carcinoma with features similar to the small cell carcinoma component in the prior oesophagectomy specimen. He was thereafter initiated on palliative chemotherapy aimed at three weekly carboplatin and etoposide aimed at a total of 4 cycles with peglasta support. Etoposide was stopped during the first cycle due to asymptomatic bradycardia. The regime was then converted to carboplatin with irinotecan for 5 cycles. Repeat computed tomographic scan performed 3 weeks after the completion of chemotherapy showed a complete response of lung and liver metastasis and no evidence of local recurrence or distant metastasis.

**Conclusion:**

The management of bipartite combined oesophageal tumours should be guided by its more aggressive component. Bipartite combined oesophageal tumours with a small cell carcinoma component are believed to demonstrate aggressive tumour biology likened to that of primary oesophageal small cell carcinoma. Preoperative confirmation of a combined tumour may be challenging, and biopsy results may only yield one of the two components. The more aggressive component is usually a small cell carcinoma, for which the mainstay of therapy is platinum-based chemotherapy rather than surgery.

## Introduction

Bipartite combined oesophageal tumours are an exceedingly rare entity. Five-year survival for squamous cell carcinomas and adenocarcinomas of the oesophagus post-curative surgery is only 20–25% [1]. Much less is known about the natural history of combined tumours of the oesophagus. The authors present a case of a bipartite combined oesophageal tumour comprising of sarcomatoid carcinoma and small cell carcinoma with early postoperative recurrence.

## Case report

**Fig. 1:**
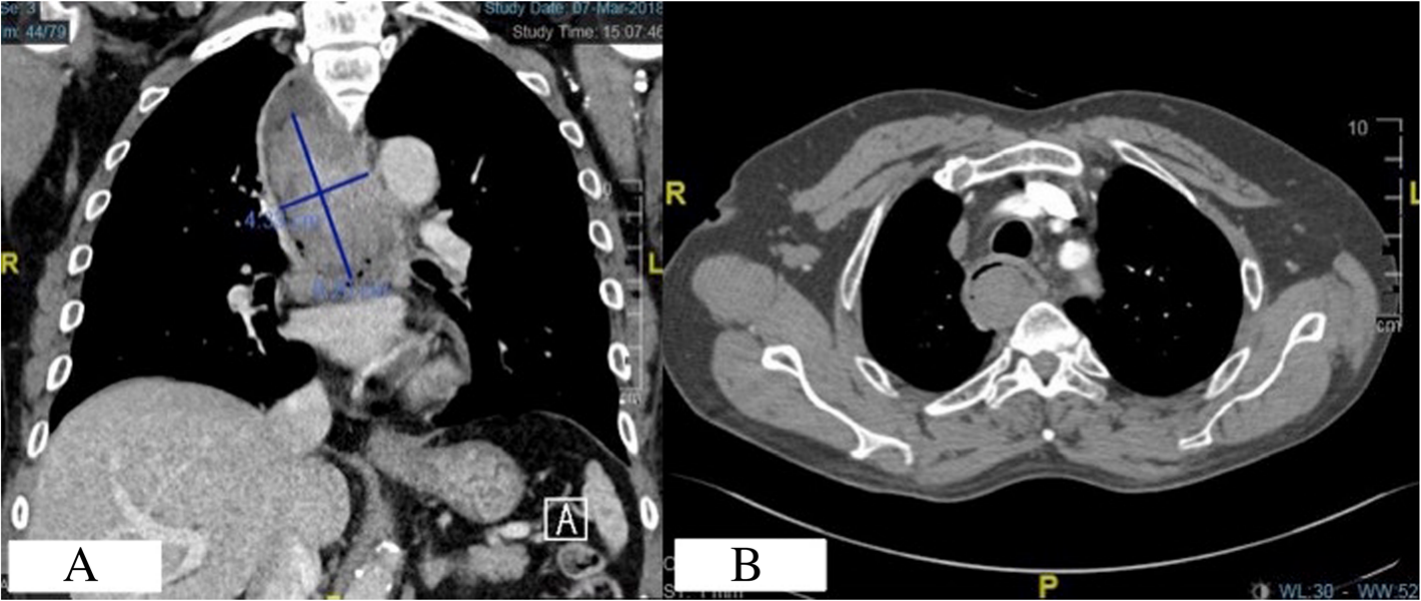
**a**, **b** Computed tomographic scan of the chest demonstrating soft tissue mass in the upper third of the oesophagus (coronal and transverse cuts)

Our patient is a 63-year-old Chinese male presenting with hemoptysis on a background of dysphagia and odynophagia for 1 month prior. He is a heavy smoker of 40 pack-years, has a history of hypertension and hyperlipidemia, and has not had any prior endoscopies. Physical examination was unremarkable. Given the presenting complaint of hemoptysis, a computed tomographic scan of his thorax was performed, revealing a polypoidal intraluminal soft tissue density in the upper third of the oesophagus (Fig. 1a, b).

**Fig. 2:**
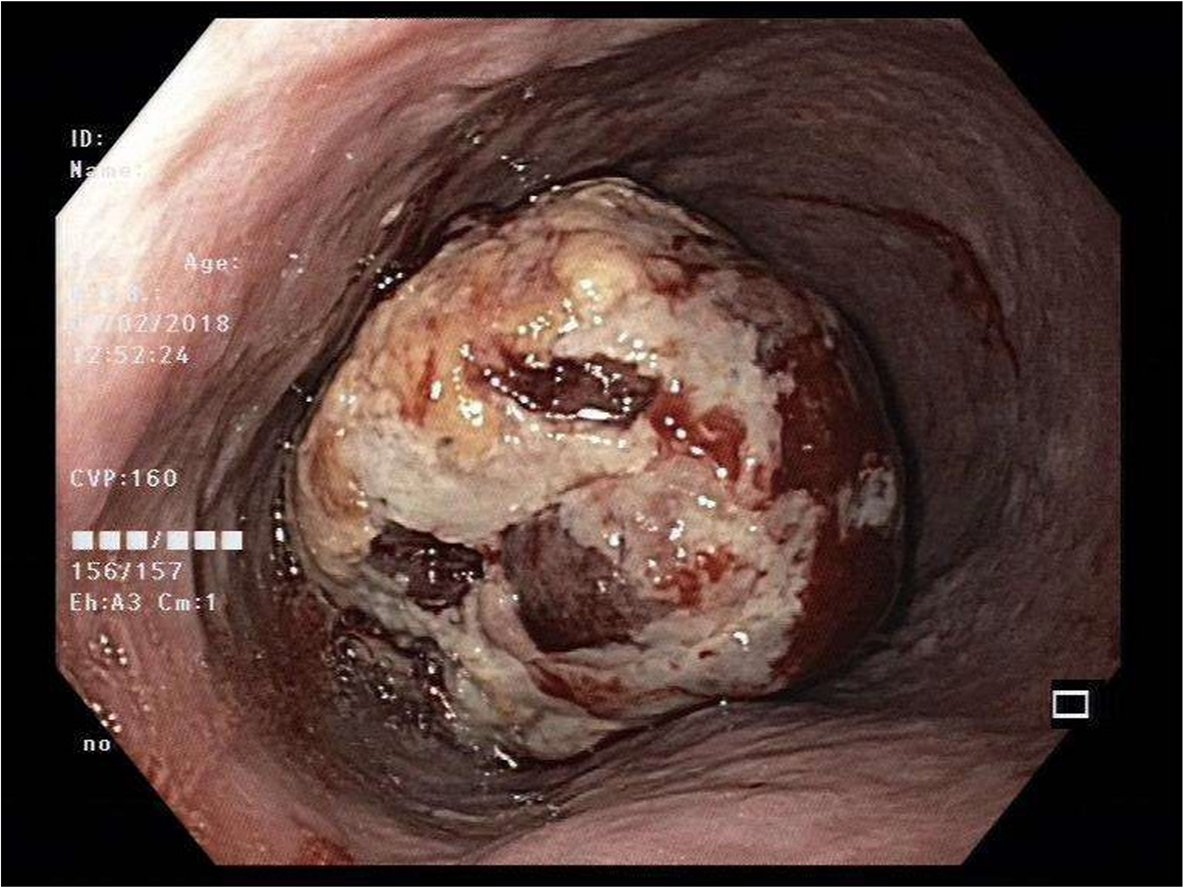
Endoscopic photograph of oesophageal tumour

An endoscopic evaluation found an exophytic oesophageal tumour with contact bleeding situated 23–30 cm from the incisors (Fig. 2). Biopsy of this lesion revealed necrotic material and fragments of tumour tissue, for which the latter composed of polygonal to spindle cells associated with a fascicular arrangement in some areas. There was also significant mitotic activity and marked nuclear pleomorphism. Immunohistochemical staining for the tumour returned negative for S-100, HMB45, AE 1/3, Cam5.2, desmin, smooth muscle actin, caldesmon, CD117 and DOG-1. The pathological conclusion for the tumour biopsy was that of a malignant high-grade tumour for which the lineage could not be established given the limited tissue. Possible differential diagnoses include sarcomatoid carcinoma and malignant undifferentiated sarcoma. Further computed tomographic scans done for the staging of the malignancy did not reveal any metastasis. Preoperative lung function tests were normal, and there was no broncho-oesophageal fistula on bronchoscopy.

**Fig. 3:**
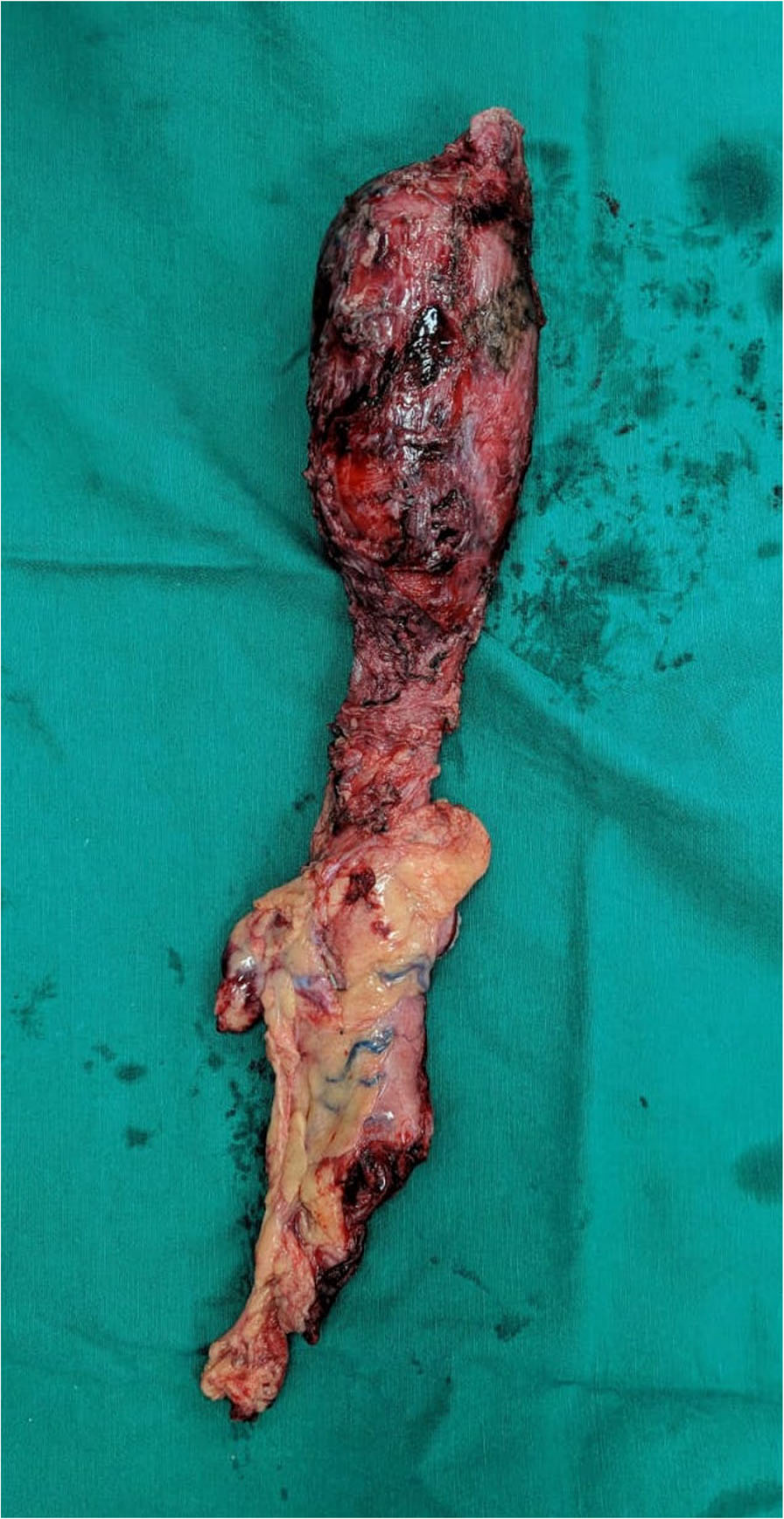
Resected specimen of minimally invasive McKeown’s oesophagectomy

With the provisional diagnosis of a high-grade oesopheageal sarcoma, the patient underwent minimally invasive McKeown’s oesophagectomy. Intraoperative findings were that of an upper oesophageal tumour (Fig. 3) without invasion into the airway or great vessels. The locoregional lymph nodes were not enlarged.

**Fig. 4:**
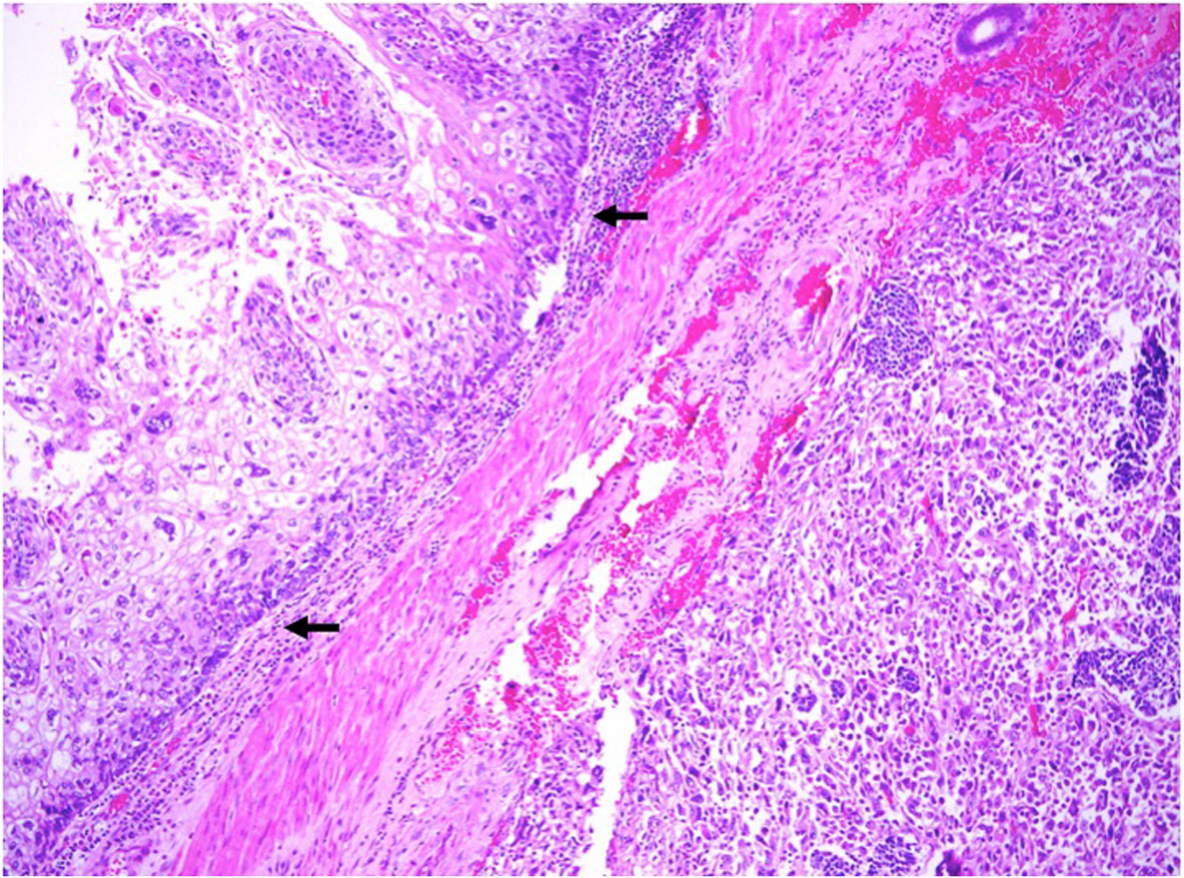
The oesophageal tumour is predominantly a sarcomatoid carcinoma composed of malignant spindle cells with overlying squamous cell carcinoma in situ (arrow). Original magnification × 100

**Fig. 5:**
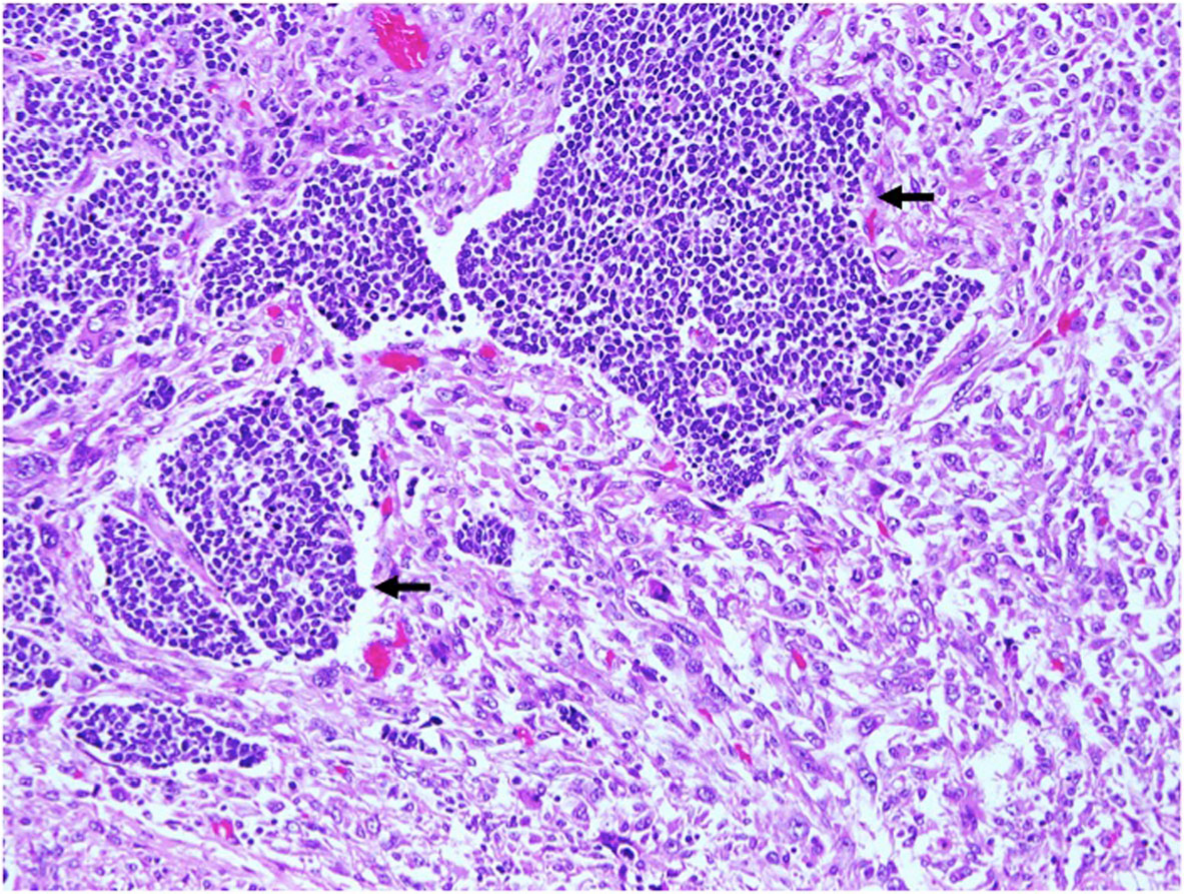
Nests of small cell carcinoma with scant cytoplasm are distributed among the malignant spindle cells with pleomorphic nuclei and ample eosinophilic cytoplasm. Original magnification × 200

**Fig. 6:**
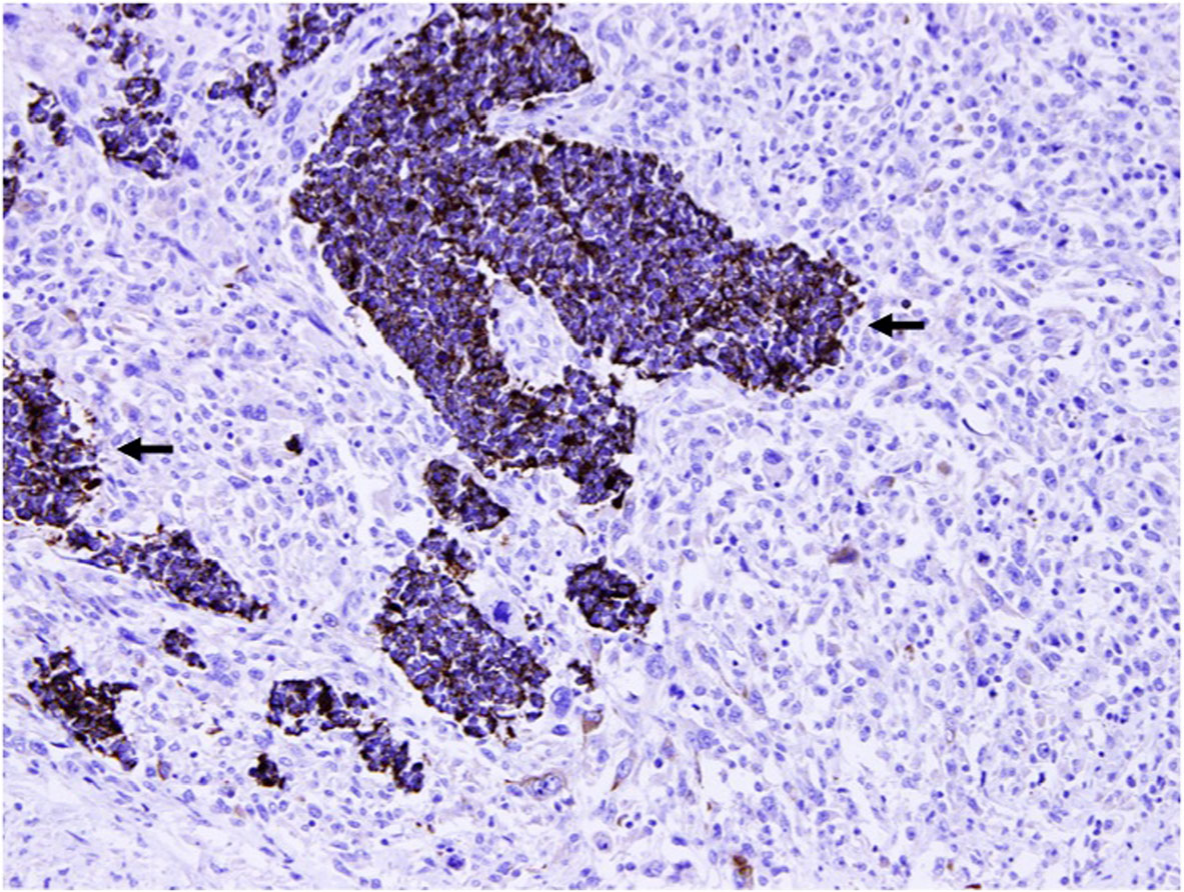
Nests of small cell carcinoma are positive for cytokeratin AE1/3 (arrows). Original magnification × 200

Examination of the specimen revealed an 11.5 × 5.3 cm polypoid mid-oesophageal tumour invading into the submucosa with clear resection margins. The tumour consisted of two histological types of malignancy within a single tumour—70% poorly differentiated spindle cell squamous carcinoma (Fig. 4) and small cell carcinoma (Fig. 5). On immunohistochemical staining, the nests of small cell carcinoma were positive for cytokeratin AE1/3 and cytoplasmic staining for the neuroendocrine marker synaptophysin. In contrast, the malignant spindle cells of the sarcomatoid carcinoma are negative for cytokeratin AE1/3 (Fig. 6). Lymphovascular invasion was negative, and none of the three lymph nodes excised were involved by malignancy. The pathological staging of the tumour was pT1bN0.

Postoperative recovery was complicated by pneumonia with a right-sided pleural effusion. The pleural effusion was drained under radiological guidance, and mediastinal collections were conservatively managed with antibiotics. He was planned for adjuvant chemotherapy in view of the small cell carcinoma component after the resolution of the postoperative infective collections.

A repeat computed tomographic scan of the thorax 3 months postoperatively to monitor for resolution of the infective collections revealed bilateral lung subcentimetre nodules. Interval repeat scan a month later demonstrated metastasis to the lung, pleura, thoracic nodes, and liver for which a biopsy of the largest lung nodule confirmed small cell neuroendocrine carcinoma. Immunohistochemical staining was positive for synaptophysin and chromogranin, with features similar to the small cell carcinoma component of the tumour in the prior oesophagectomy specimen. There were no squamous or spindle cell components seen in the lung biopsy. He had not received the intended adjuvant chemotherapy regime, and in light of the new metastasis, he was initiated on palliative chemotherapy aimed at three weekly carboplatin and etoposide aimed at a total of 4 cycles with peglasta support. Etoposide was stopped during the first cycle due to asymptomatic bradycardia. The regime was then converted to carboplatin with irinotecan for 5 cycles. Repeat computed tomographic scan performed 3 weeks after the completion of chemotherapy showed a complete response of lung and liver metastasis and no evidence of local recurrence or distant metastasis.

## Discussion

Combined tumours are part of an uncommon group of neoplasms that consist of more than one cell population. Other similar types of neoplasms include collision tumours and composite tumours. Collision tumours are inherently different from combined tumours in that they have two distinct cell populations originating from topographically separate sites, developing in juxtaposition without any or only minimal areas of intermingling [2, 3]. On the other hand, composite tumours which are characterised by two divergent lineages originate from the same neoplastic clonal proliferation [4].

The majority of oesophageal combined carcinomas are associated with small cell carcinoma, and a combination with squamous cell carcinoma is the most common [2]. It is postulated that these combined tumours arise from pluripotent cells present in the squamous epithelium or ducts of the submucosal glands, leading to a heterogenous differentiation within a single tumour [5–7]. According to the World Health Organization histological classification of tumours of the digestive system [8], oesophageal tumours can be broadly divided into epitheal, non-epithelial and secondary tumours, for which both spindle cell (squamous) carcinoma and small cell carcinoma are distinct entitites under epithelial tumours. Spindle cell carcinomas, also coined carcinosarcomas, are a rare variant of squamous cell carcinomas with a sarcomatoid spindle cell component. Macroscopically, these tumours demonstrate a polypoid growth pattern. On microscopic examination, most specimens show a gradual transition between carcinomatous and sarcomatous components [8].

Small cell carcinomas are considered to be poorly differentiated endocrine carcinomas and are described to be indistinguishable from its counterpart in the lung in terms of histological, immunohistochemical and clinical features [9]. Given the rarity of the combined oesophageal tumours, the biological behaviour of these neoplasms is not well established. Previously, Tadashi et. al [10] described that the small cell carcinoma component of combined oesophageal tumours confers extremely aggressive tumour biology and suggest that these tumours be managed as per primary small cell carcinomas.

In our patient, an endoscopically acquired biopsy of the tumour was that of a malignant high-grade tumour of unknown lineage, while imaging suggested a sarcomatoid carcinoma or malignant undifferentiated sarcoma.

Initial management of the patient was based on that of an undifferentiated sarcoma. Despite resection with clear magins, our patient unfortunately developed early metastatic recurrence just 4 months postoperatively. Oesophageal sarcomas are extremely rare, and literature pertaining to their management is limited. A preoperative PET scan was not performed as it is not the standard of care for sarcomas [11]. En bloc oesophagectomy with radical lymphadenectomy is the recommended option for oesophageal sarcomas and is associated with a significant survival advantage [12]. Nonetheless, long-term survival tends to be poor with a high rate of local and metastatic recurrance [13].

In a small case series of three combined oesophageal tumours, Tadashi et al. [10] described the aggressive nature of the tumour with all three patients developing metastatic tumour recurrence soon after oesophagectomy and subsequent demise. Two of the three patients received postoperative adjuvant chemotherapy with a cisplatin-based regime. Metastatic recurrence was diagnosed 1 to 10 months postoperatively. Similar to our patient, the histology of the metastatic deposits in lymph nodes and skin was small cell carcinoma—supporting the postulation that the small cell component was responsible for its aggressive behaviour.

Another case series of two combined oesophageal tumours [2] demonstrates the poor prognosis of tumours with small cell component. These cases had confirmed small cell component on initial endoscopic biopsy, and thus, surgery was not considered. Both were stage II disease and received cisplatin-based chemotherapy and radiation but died of metastatic disease 7 and 15 months, respectively, after presentation. Hosokawa et al. [14] described five patients who underwent only oesophagectomy with curative intent, and all patients developed early relapse with a median survival of 7 months.

Postoperative 5-year survival rate was significantly lower in patients with small cell carcinoma compared to patients with squamous cell carcinoma, and the presence of lymph nodal metastasis was a significant determinant [15]. Recurrence in resected primary oesophageal cancers of either squamous cell carcinoma or adenocarcinoma was detected in about 45 to 50% of patients within the first postoperative year [16–18], and the median time to developing recurrence was 12 months with no difference between squamous cell carcinoma and adenocarcinoma [16]. This is in contrast to patients with oesophageal small cell carcinoma who have a median survival time of 11–18 months following surgery [18, 19]. Situ et al. [18] found that regional lymph node involvement was the only significant prognostic factor for survival after surgical resection of oesopahgeal small cell carcinoma.

Primary oesophageal small cell carcinoma itself is a rare entity, and there are no strong recommendations for the optimal treatment regime. At present, treatment for localised disease is centred around systemic chemotherapy with the consideration of local treatment such as radiotherapy. The platinum-based chemotherapy regime would consist of etoposide plus platinum such as cisplastin or carboplatin—which is also the standard regimen for small cell carcinoma of the lung [2, 20–24]. Chemotherapy is well-established for the management of small cell lung cancer [25, 26], and extra-pulmonary small cell carcinoma is chemosensitive as well [27, 28]. Chemotherapy alone is recognised to improve survival in oesophageal small cell carcinoma [29], with further survival benefit with concurrent chemoradiotherapy for limited disease [15, 30, 31]. With the high frequency of early systemic relapse after local treatment and chemosensitivity of extra-pulmonary small cell carcinoma, chemotherapy has been the cornerstone of treatment of oesophageal small cell carcinoma. Our patient was also initiated on the same chemotherapy regimen.

For patients with metastatic small cell carcinoma of the oesophagus, small studies with limited data have shown longer survival with palliative chemotherapy [20, 23, 25]. On the other hand, oesophagectomy or radiotherapy alone is not recommended in insolation due to poor outcomes and should be combined with adjuvant or neoadjuvant platinum-based chemotherapy [20, 22]. The median survival of primary small cell carcinoma is about 8 months in patients with limited disease and 3 months in patients with extensive disease [22].

## Conclusion

The management of bipartite combined oesophageal tumours should be carried out in accordance with its more aggressive component. Bipartite combined oesophageal tumours with a small cell carcinoma component are believed to possess aggressive tumour biology likened to that of primary oesophageal small cell carcinoma. Preoperative confirmation of a combined tumour may be challenging, and biopsy results may only yield one of the two components. The more aggressive component is usually a small cell carcinoma, for which the mainstay of therapy is platinum-based chemotherapy rather than surgery.
